# Decoding the pancreatic cancer microenvironment: The multifaceted regulation of microRNAs

**DOI:** 10.1002/ctm2.70354

**Published:** 2025-06-27

**Authors:** Jie Ji, Dandan Jin, Junpeng Zhao, Xudong Xie, Yujie Jiao, Xuyang He, Yuxuan Huang, Lirong Zhou, Mingbing Xiao, Xiaolei Cao

**Affiliations:** ^1^ Department of Gastroenterology Affiliated Hospital of Nantong University Medical School of Nantong University Nantong Jiangsu China; ^2^ Department of Gastroenterology Affiliated Changshu Hospital of Nantong University Changshu Jiangsu China; ^3^ Department of Clinical Medicine Medical School of Nantong University Nantong Jiangsu China; ^4^ Department of Pathology Medical School of Nantong University Nantong Jiangsu China

**Keywords:** exosome, immune microenvironment, miRNA, PC, tumour microenvironment

## Abstract

Pancreatic cancer (PC) is an extremely deadly type of cancer, and the 5‐year survival rate remains less than 10%. The tumour microenvironment (TME) affects the occurrence, progression and treatment outcomes of PC. MicroRNAs (miRNAs) are essential to regulate PC TME. This review delves into the different roles of miRNAs in the PC TME, including exosome communication, angiogenesis, interactions with cancer‐associated fibroblasts, the immunological and neuronal microenvironments and metabolic reprogramming. However, research on the complex regulatory networks and synergistic effects of miRNAs in the TME is still insufficient, and their clinical translation and application face challenges. This review summarised the activities of miRNAs in the PC TME, guiding future research and therapeutic strategies involving miRNAs in PC. Future studies should integrate advanced technologies to decode the spatiotemporal dynamics of miRNA regulation within the TME and develop optimised nanodelivery systems for stable and targeted miRNA delivery, advancing clinical applications in PC treatment.

## Graphical abstract

miRNAs play multiple roles in the tumour microenvironment of pancreatic cancer, including angiogenesis, interactions with cancer‐associated fibroblasts, the immune microenvironment, exosome communication, the neural microenvironment and metabolic reprogramming.

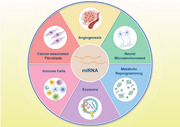

## 1 INTRODUCTION

Because of the high fatality rate and dismal prognosis, PC is commonly referred to as the ‘king of cancers’. The prevalence and death rates of PC continue to climb worldwide, and the 5‐year survival rate remains below 10%.^1^, ^2^ Because the PC's onset is insidious and early symptoms lack specificity, the majority of patients are diagnosed at an advanced stage, missing the opportunity for a surgical cure. Chemotherapy is a common treatment method, but its effectiveness in treating PC is not satisfactory.^3^ In recent years, immunotherapy and targeted therapy have made some progress in the field of cancer treatment, but owing to the complexity of the PC TME, the effectiveness of these treatments is restricted.^4^, ^5^ Therefore, a more thorough comprehension of the features of PC TME becomes essential for discovering more efficient methods of treatment.

**FIGURE 1:**
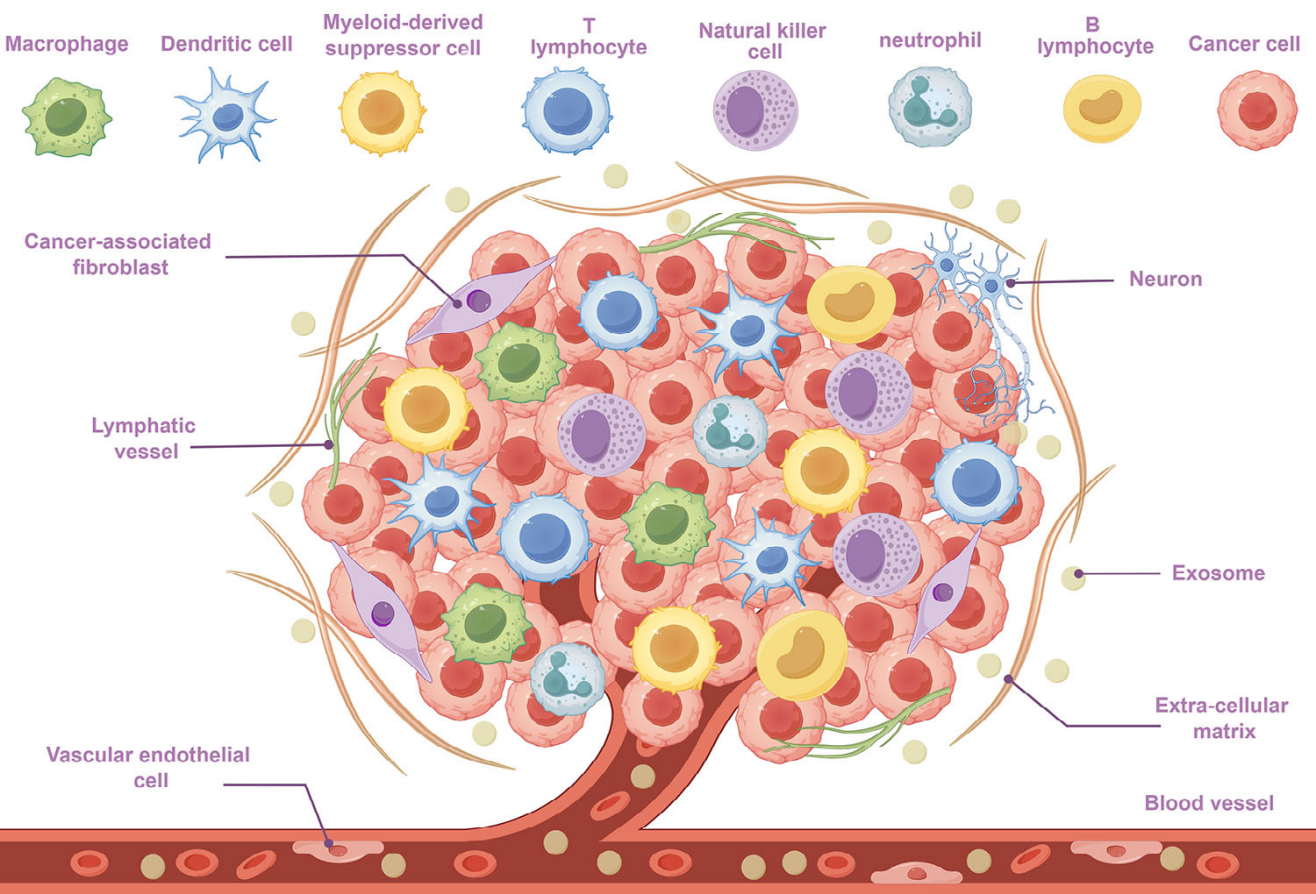
Composition of the TME. The TME comprises diverse immune cell populations including macrophages, dendritic cells (DCs), myeloid‐derived suppressor cells (MDSCs), T lymphocytes, natural killer (NK) cells, neutrophils and B lymphocytes, stromal elements including CAFs, vascular endothelial cells and structural components including lymphatic vessels, neurons, exosomes, the extracellular matrix and blood vessels, which collectively contribute to immunosuppressive and tumour‐promoting niche characteristics.

Tumour cells, different non‐tumour biological components, and the extracellular matrix make up the complex ecology known as the TME. In spite of tumour cells, the TME contains cancer‐associated fibroblasts (CAFs), immune cells, vascular endothelial cells, nerve cells, matrix components, metabolic factors and cellular secretions (e.g., cytokines, exosomes and metabolites) (Figure 1).^6^ The interactions among these components not only encourage PC growth and metastasis but also enhance the chemoresistance and immune escape capabilities of malignant cells by altering the TME.^7^ The stroma‐rich and collagen‐dense architecture of the PC TME, coupled with its hypoxic and immunosuppressive features, poses challenges for treatment while also providing new perspectives for studying its mechanisms of action.^8^, ^9^

A category of non‐coding RNAs having a length roughly 18–25 nucleotides is known as miRNAs. By binding themselves to target mRNAs’ 3′UTR (3′ untranslated region), they control the expression of genes.^10^ Some non‐coding RNAs, including long non‐coding RNA (lncRNA) and circular RNA (circRNA), can act as ‘sponges’ for miRNAs by providing multiple binding sites for miRNAs. This binding prevents miRNAs from interacting with target mRNAs, thus removing miRNAs’ inhibitory action on target genes, and this mechanism is known as the sponge effect.^11^ In PC, improper levels of miRNA is strongly associated with the cancer's occurrence, progression, medication resistance and unfavourable prognosis.^12^ The possibility of miRNAs as tumour diagnostic and treatment targets is progressively being investigated due to their great stability and simplicity of detection.^13^

miRNAs play an increasingly important role in the TME, according to researchers. They govern the intrinsic characteristics of tumour cells and additionally impact tumour development through the dynamic regulation of various aspects of the TME. Through controlling the growth, migration and differentiation of vascular endothelial cells, miRNAs have a direct impact on the blood supply of malignancies.^14^, ^15^ They also regulate the activation state and secretory function of CAFs, altering the extracellular matrix and increasing tumour cells’ ability to invade.^16^ In the immunological milieu, miRNAs manage the immune escape capability of tumours by managing the differentiation, activation and effectiveness of immune cells.^17^, ^18^ Additionally, as important components of exosomes, miRNAs participate in intercellular signalling, impacting cancer cell invasion and migration.^14^ miRNAs enhance neural invasion capabilities in the neural microenvironment by regulating interactions among tumours and nerves, providing a significant function on pain and distant metastasis caused by PC.^19^ In metabolic reprogramming, miRNAs help tumour cells adapt to the hypoxic and nutrient‐deprived TME by regulating key metabolic enzymes and metabolic signalling pathways, providing support for tumour cell growth.^20^

This review provides a systematic summary of miRNAs’ function in the PC TME and explores six aspects: exosomes, angiogenesis, CAFs, the immune microenvironment, the neural microenvironment and metabolic reprogramming. By integrating these studies, we aim to further reveal the mechanisms of action of miRNAs in the PC TME, providing a new theoretical basis for the establishment of precise diagnostic and treatment procedures for PC.

## 2 EXOSOMAL miRNA REGULATION OF THE PC TME

The past few decades have witnessed tremendous progress in the investigation of PCs because of multiple roles that exosomes play. Exosomes are nanoscale (diameter 30–150 nm) extracellular vesicles commonly seen in human fluids like milk, urine and blood.^21^, ^22^ Exosomes are considered essential agents of interactions between cells and can transport bioactive chemicals, such as lipids, proteins and nucleic acids. The miRNAs in exosomes are involved in modulating tumour formation, treatment resistance and immunological responses but also demonstrate significant potential in the early detection and precise treatment of diseases.^23^

### 2.1 Research on exosomal miRNAs as PC biomarkers

Investigation of exosomal miRNAs has gradually revealed their important roles in the early diagnosis, staging, prognosis assessment and disease mechanisms of PC (Table 1). A number of investigations have shown that PC patients exhibit markedly elevated levels of miR‐1246, miR‐4644, miR‐3976, miR‐4306, miR‐191, miR‐21 and miR‐451a in their serum exosomes.^24^ The high enrichment of miR‐196a and miR‐1246 in PC patients’ plasma exosomes shows their diagnostic value for localised PC.^25^ Similarly, PC patients’ serum exosomes have far greater levels of miR‐451 than healthy subjects, whereas the miR‐720 levels are reduced, indicating high sensitivity and specificity, which also helps diagnose PC early.^26^ Exosomes produced from plasma that contain miR‐18a and miR‐106a may also be useful in the PC diagnosis.^27^ Furthermore, miRNAs carried by exosomes in other body fluids have an enormous effect on PC diagnosis. The miR‐3940‐5p/miR‐8069 ratios in urine exosomes and markers such as miR‐21, miR‐25, miR‐16 and miR‐210 in pancreatic juice exosomes have expanded the application scenarios of liquid biopsy and further improved diagnostic sensitivity and specificity through combined detection with CA19‐9.^28^, ^29^ The quantity of miR‐20a in duodenal juice exosomes is greater in PC patients compared with healthy individuals, providing an innovative biomarker of PC early detection.^30^ Notably, miR‐19b's diagnostic utility in exosomes generated from plasma is preferable to the CA19‐9, which is already used in clinical practice.^31^

**TABLE 1:** Exosomal miRNAs as biomarkers for PC.

miRNA	Materials	Status	Biomarker	Sensitivity	Specificity	AUC	References
1246 4644 3976 4306	Serum	Up	Diagnosis	0.81	0.94		41
191	Serum	Up	Diagnosis	0.719	0.842	0.788	24
21			Diagnosis Prognosis	0.811	0.81	0.826	
451a			Diagnosis	0.857	0.736	0.759	
1246	Plasma	Up	Early diagnosis			0.73	25
196a						0.81	
451	Serum	Up	Diagnosis			0.933	26
720		Down				1	
3940‐5p/8069 ratio	Urine	Up	Diagnosis	0.581	0.892	0.732	28
21	Pancreatic juice	Up	Diagnosis			0.64	29
25						0.63	
16						0.64	
210	Serum					0.62	
20a	Duodenal drainage fluid	Up	Diagnosis			0.88	30
18a 106a	Plasma	Up	Diagnosis				27
19b	Plasma	Down	Diagnosis	0.855	0.906	0.942	31
21	Serum	Up	Diagnosis Prognosis			0.869	32
210						0.823	
484‐3p	Serum	Up	Diagnosis Prognosis			0.69	33
205‐5p	Plasma	Down	Diagnosis Prognosis			0.829	34
1293	Tissue	Up	Prognosis				35

Apart from the diagnostic usefulness, exosomal miRNAs’ potential for PC staging and prognostic evaluation has also been confirmed. Research indicated that circulating exosomal miR‐21 and miR‐210 are intimately associated with the prognosis and status of PC patients.^32^ miR‐483‐3p is overexpressed when PC is only getting started, with its levels significantly correlated with patient prognosis.^33^ Similarly, tumour development and the patient's state of survival are correlated with the amount of miR‐205‐5p in plasma exosomes from Brazilian PC patients.^34^ miR‐1293 in exosomes is also a prognostic biomarker for PC.^35^ Besides, 24 miRNA markers including miR‐144‐5p, miR‐3148 and miR‐3133, have been found in exosomes and could be useful biomarkers for diabetes brought on by PC.^36^

In summary, miRNAs from different body fluids have unique characteristics as PC biomarkers. Plasma and serum exosomal miRNAs, with their high abundance and well‐established detection methods, have become a research focus, with some markers even outperforming the traditional marker CA19‐9 in diagnostic efficacy. However, they may be subject to interference from systemic factors.^37^ Urinary exosomal miRNAs are non‐invasive and highly stable but are present at relatively low concentrations, necessitating high‐sensitivity detection techniques.^38^ Exosomal miRNAs in pancreatic juice and duodenal fluid can directly reflect the local pancreatic microenvironment and have stronger specificity, but their collection is more inconvenient, and they are susceptible to the effects of digestive fluids.^39^ In terms of clinical translation, an ideal biomarker should possess high sensitivity and specificity. However, currently, individual exosomal miRNAs rarely meet the ideal standards required for clinical application; for example, their sensitivity and specificity both exceed 90%. Nevertheless, studies have shown that combining multiple exosomal miRNAs or integrating them with other markers can significantly enhance diagnostic effectiveness. Technologically, breakthroughs in single‐vesicle analysis have significantly improved the sensitivity of biomarker detection. For example, total internal reflection fluorescence imaging technology can be used to measure miR‐21 in individual exosomes in serum quantitatively, with diagnostic performance superior to that of traditional PCR and supporting therapeutic monitoring. This provides a new paradigm for detecting low‐abundance miRNAs.^40^ Future research should integrate the characteristics of miRNAs from different body fluids, explore more miRNA combinations with high diagnostic value and verify their performance through large‐scale clinical trials while leveraging new technologies such as single‐vesicle analysis to increase detection sensitivity.

### 2.2 Research on exosomal miRNAs’ multifaceted involvement in PC

Numerous investigations have revealed that PC cells can directly influence the progression, drug resistance and metabolic disorders through exosomes carrying specific miRNAs.^42^ In terms of tumour growth, miR‐222 in exosomes of malignant cells encourages the proliferation via modulating the level and location of p27, and the level of miR‐222 is connected to TNM stage and tumour size.^43^ By specifically targeting ZNF689, miR‐339‐5p in exosomes is markedly down‐regulated, preventing PC cells from invading and migrating.^44^ Furthermore, it has been established that miR‐1246 and miR‐1290 in tumour cell exosomes encourage the pancreatic stellate cells’ (PSCs) activation, hence facilitating the advancement of PC.^45^

In the context of drug resistance, exosomes released by PC cells deliver miR‐155 to down‐regulate the expression of the drug metabolism enzyme DCK and increase the activity of the antioxidant enzymes SOD2 and CAT, thereby increasing the resistance of PC to gemcitabine (GEM).^46^ Similarly, miR‐31‐5p in exosomes that generated by PC cells enhances the GEM resistance via modulating the LATS2–Hippo signalling pathway and stimulating SPARC secretion.^47^ However, the enrichment of miR‐1976 in PC patients’ exosomes enhances their sensitivity to chemotherapeutic drugs by regulating the proapoptotic gene XAF1, suggesting a new strategy to increase chemotherapy's efficacy.^48^ In the investigation of radioresistance, exosomal miR‐6855‐5p released from cancer cells stimulates epithelial–mesenchymal transition (EMT) by inhibiting FOXA1, thereby enhancing the PC's radioresistance, indicating that it may be a biomarker for radiation sensitivity.^49^ Radiotherapy‐induced apoptotic tumour cells release exosomes containing miR‐194‐5p, which boosts the DNA damaging of tumour‐repopulating cells, thereby promoting their survival and repopulation and affecting radioresistance.^50^

In metabolic disorders, exosomes released from PC cells include miR‐let‐7b‐5p, which targets RNF20 to turn on the STAT3/FOXO1 axis and increases insulin resistance, revealing its potential role in PC‐related metabolic abnormalities.^51^ Exosomal miR‐16‐5p and miR‐29a‐3p from cancer exosomes regulate fat metabolism, which may be closely related to cachexia caused by PC.^52^ In addition, by blocking ADCY1 and EPAC2, miR‐19a in PC cells’ exosomes disrupts pancreatic β‐cell function, offering fresh perspectives on the aetiology of diabetes linked to PC.^53^

Additional research has demonstrated that exosomal miR‐125a‐5p from PC cells promotes osteoclast differentiation through the TNFRSF1B signalling pathway, suggesting a new molecular mechanism for understanding bone loss caused by PC.^54^ Furthermore, miR‐21 in exosomes from cancer cells promotes JNK‐dependent muscle cell apoptosis by attaching itself to muscle cells’ TLR7 receptor, which could be crucial in the atrophy of muscles brought on by PC.^55^

### 2.3 Other research on exosomal miRNAs in PC

**FIGURE 2:**
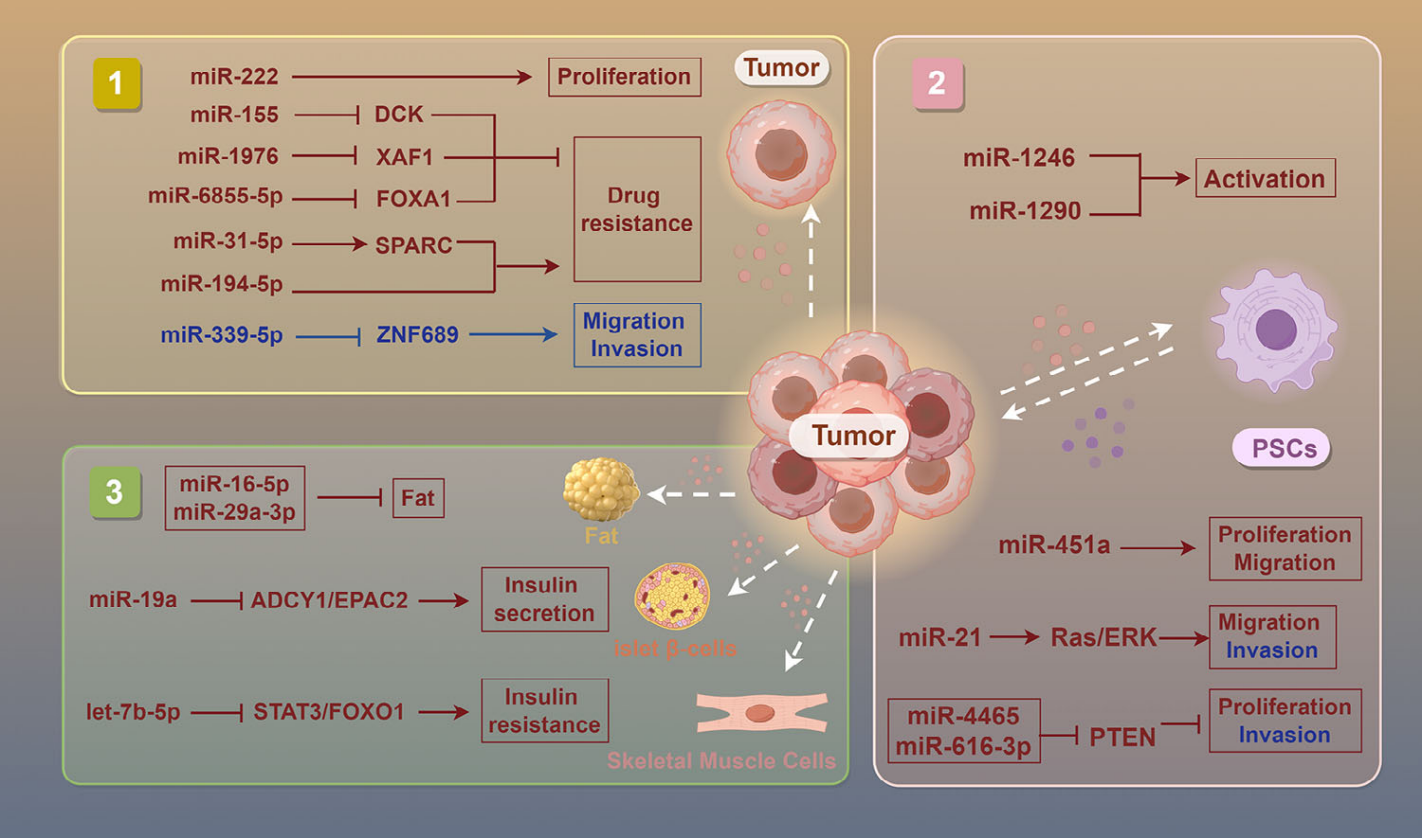
Exosomal miRNA regulation of the TME in PC. (A) Tumour‐derived exosomal miRNAs modulate the PC cells malignant behaviours and drug resistance of PC cells. (B) Exosomal miRNAs carried by PC cells promote the activation of PSCs. Communication between PSC‐derived exosomal miRNAs and tumour cells plays a key role in PC progression. (C) PC cell‐derived exosomal miRNAs contributing to fat atrophy, diabetes and bone loss caused by PC. Red text indicates miRNAs that promote PC progression, whereas blue text denotes miRNAs that suppress tumour development.

Research has shown that PSCs play crucial roles in the occurrence and progression for PC through intercellular communication mediated by miRNAs in exosomes. First, exosomes secreted by PSCs are rich in miR‐451a, which PC cells can absorb, encouraging their motility, proliferation and chemokine gene activation.^56^ Similarly, the PC cells’ markedly enhanced capacity to migrate and invade seems very similar with the raised level of miR‐21 in PSC exosomes. miR‐21 stimulates the Ras/ERK signalling cascade, induces EMT and enhances MMP2 and MMP9, driving tumour invasion and metastasis.^57^, ^58^ In hypoxic environments, miR‐4465 and miR‐616‐3p in PSC exosomes stimulate the AKT signalling pathway, which boosts PC cells’ proliferation and invasion.^59^ In addition, miR‐4443 and miR‐3909 have been found to regulate the HIF1A and KIF13A genes, possibly assisting PC cells in their development and survival by modulating intracellular vesicle transport (Figure 2).^60^

In conclusion, exosomal miRNAs, as key carriers of intercellular communication, have demonstrated multifaceted regulatory capabilities in PC's incidence, progression and management. They not only influence tumour development, invasion and medication resistance through regulating gene expression and signalling pathways but also strongly influence metabolic disorders and the shaping of the TME. However, current research still faces the bottleneck of exosome heterogeneity. Future studies should integrate single‐vesicle multiomics analysis and spatial imaging techniques to achieve precise dissection of exosome subpopulations, thereby providing new perspectives for the future precision PC treatment.

## 3 miRNA MODULATION OF ANGIOGENESIS IN THE PC TME

The act of creating fresh vessels from current ones is known as angiogenesis, and it is necessary for the development of malignancies. Cancer cells stimulate new blood vessels formation by producing proangiogenic factors such as VEGF and ANG2, thereby meeting the demands for oxygen and nutrients and promoting tumour growth.^61^ Angiogenesis provides avenues for tumour cell metastasis in addition to promoting tumour growth. The abnormal structure of tumour neovessels (such as irregularity and high permeability) increases the chances of tumour cells entering the bloodstream, thereby facilitating metastasis. According to research, tumour angiogenesis is inextricably tied to metastasis formation and that metastatic tumours are often accompanied by increased angiogenesis.^62^, ^63^ Additionally, angiogenesis is crucial for tumour immune evasion. Tumour neovessels promote the accumulation of immunosuppressive cells, inhibiting the anti‐tumour immune response.^64^ Research has shown that multiple signalling pathways, including the VEGF/VEGFR, angiopoietin/Tie2 and FGF pathways, are involved in regulating tumour angiogenesis.^65^ Activation of these pathways leads to the proliferation and migration of endothelial cells, thereby promoting angiogenesis.

miRNAs play important roles in regulating the expression of genes related to angiogenesis.^66^ Zhang et al.^67^ reported that miR‐223 promotes the angiogenesis and tumour formation of PC cells via inhibiting SLC4A4. MiR‐301a enhances the angiogenic capacity of tumours in PC by focusing on SOCS5, which encourages PC metastasis.^68^ The miR‐24–Bim pathway is also important for increasing tumour angiogenesis, apoptosis and tumour cell proliferation.^69^ In addition, miR‐27a is crucial for the angiogenesis of PC; it targets and inhibits BTG2, affecting the function of HMVECs and thereby promoting tumour angiogenesis.^70^ miR‐27a can also promote angiogenesis by directly targeting the angiogenesis inhibitor SEMA6A, thus affecting tumour growth and metastasis.^71^ Furthermore, miR‐139 and miR‐200c can promote PC angiogenesis by influencing the formation of tubular structures during angiogenesis.^72^

The role of miRNAs in inhibiting angiogenesis additionally indicates promise in the PC treatment. The VEGF/VEGFR axis constitutes a central pathway regulating angiogenesis, driving neovascularisation by promoting endothelial cell proliferation, migration and survival. miRNAs suppress tumour‐associated angiogenesis through direct targeting of VEGF/VEGFR or indirect modulation of downstream effectors. miR‐29b‐3p^73^ and the miR‐200^74^ family directly target and inhibit VEGF, thereby suppressing angiogenesis in PC. miR‐29b,^75^ miR‐497,^76^ miR‐26a^77^ and miR‐141^78^ suppress angiogenesis by inactivating the VEGF/VEGFR pathway through down‐regulating ROBO1 and SPGAP2, Twist, E2F7 and TM4SF1, respectively. Additionally, miR‐410 exerts its effects by targeting AGTR1 to inhibit angiotensin II‐induced VEGF expression.^79^ By controlling the CDKN2D/VEGF axis, miR‐451a prevents PC from growing and from angiogenesis.^80^ miR‐214‐3p^81^ suppresses angiogenesis by targeting YAP1 to modulate the VEGF signalling pathway. Moreover, miRNAs can also inhibit angiogenesis by blocking key signalling pathways; miR‐454 inhibits angiogenesis via decreasing LRP6 and blocking the signalling pathway of Wnt/β‐catenin,^82^ and miR‐206 inhibits the NF‐κB pathway via inhibiting K‐Ras.^83^ In addition, miRNAs can inhibit tumour angiogenesis by targeting other key angiogenic factors. For example, miR‐34a^84^ and miR‐145^85^ significantly suppress angiogenesis by down‐regulating CCL5, bFGF and Ang‐2. miR‐330‐5p,^86^ miR‐520a‐5p,^87^ miR‐590‐5p,^88^ miR‐34a‐3p and miR‐34a‐5p^89^ further exert regulatory functions by targeting other factors related to angiogenesis. Some miRNAs are also affected by their target gene suppression effects through sponge adsorption by lncRNAs and circRNAs, thereby affecting angiogenesis. The lncRNA NORAD may regulate the activity of miR‐532‐3p through a competitive endogenous RNA (ceRNA) way, altering the Nectin‐4 level and affecting PC angiogenesis and cell proliferation.^90^ MiR‐1179,^91^ miR‐218‐5p,^92^ and miR‐557^93^ target RHPN2, PPME1 and PDL1, respectively, to inhibit PC angiogenesis and progression, whereas circ_0000284, circ_0014784, circ_0050102 and circ_0058058 can inhibit the corresponding miRNAs through a ‘sponge effect’. In summary, these studies reveal that miRNAs regulate angiogenesis in PC through direct or indirect means, providing new molecular targets and strategies for anti‐angiogenic therapy.

**FIGURE 3:**
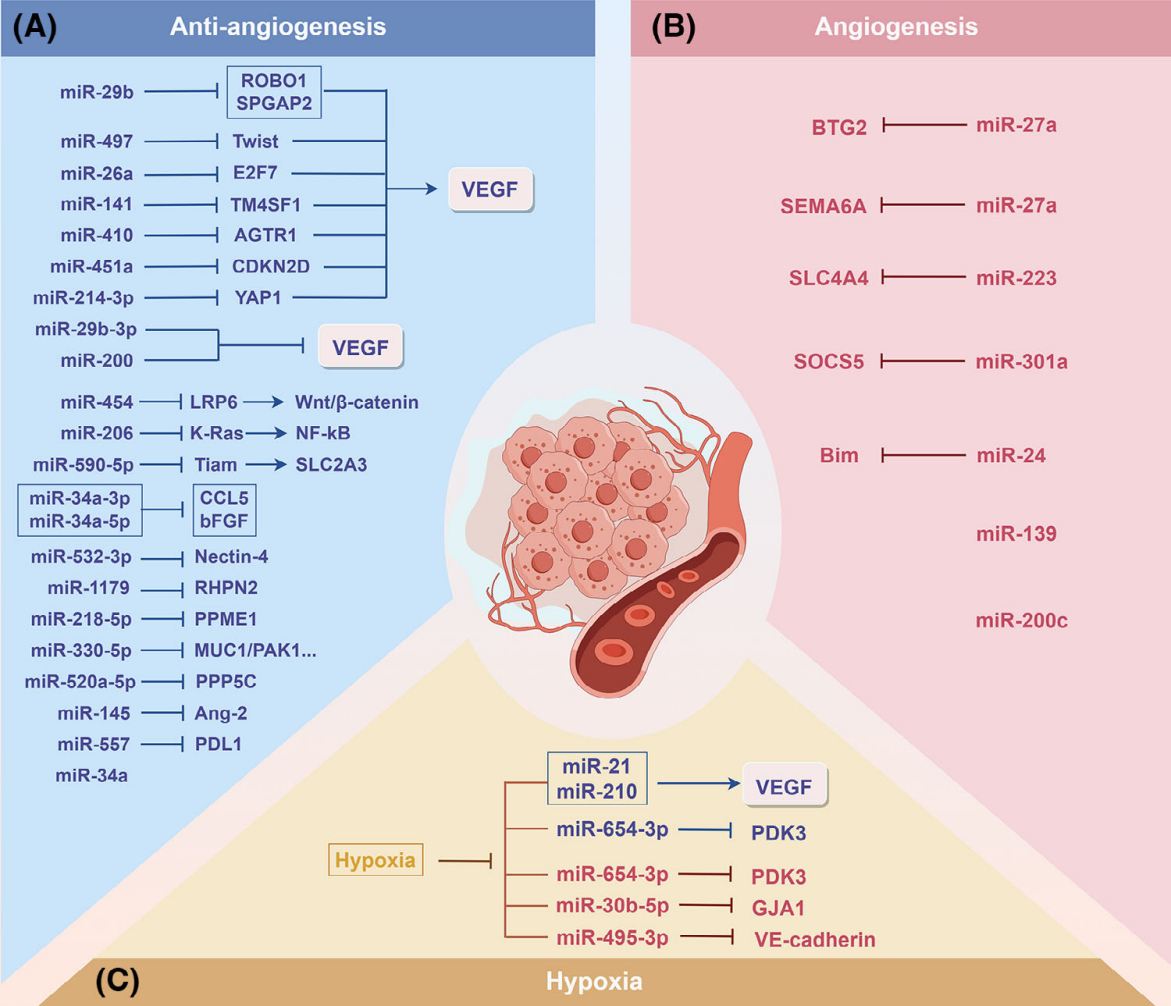
miRNA modulation of angiogenesis in PC. Tripartite regulatory mechanisms of miRNAs governing angiogenesis include anti‐angiogenic inhibition (A), proangiogenic activation (B) and hypoxia‐induced vascular remodelling (C) in the TME of PC. Red text indicates miRNAs that promote PC progression, whereas blue text denotes miRNAs that suppress tumour development.

Hypoxia, an important characteristic of the TME of PC, has a significant effect on tumour angiogenesis. Under hypoxic conditions, PC cells regulate miRNAs through various pathways, thereby affecting angiogenesis. Under hypoxic conditions, miR‐30b‐5p derived from pancreatic ductal adenocarcinoma cells significantly promotes angiogenesis by inhibiting GJA1.^94^ In addition, under hypoxic conditions, UCA1 produced from PC cells competes with miR‐96‐5p, initiates the ERK1/2 signalling pathway, thereby promoting angiogenesis in vascular endothelial cells.^95^ Under hypoxic conditions, miRNAs can also be regulated by lncRNAs and circRNAs to affect angiogenesis. After HIF‐1α and EIF4A3 up‐regulate the level of circRNF13, circRNF13 inhibits miR‐654‐3p through a sponge effect, increasing PDK3 and so boosting angiogenesis and malignancy in PC.^96^ In addition, under hypoxic conditions, NORAD directly binds to miR‐495‐3p, enhancing VM (vasculogenic mimicry).^97^ On the other hand, CDF inhibits the levels of miR‐21 and miR‐210, reducing the synthesis of VEGF and IL‐6, thereby effectively reducing angiogenesis and tumour invasiveness under hypoxic conditions (Figure 3).^98^, ^99^, ^100^, ^101^ These studies show that under hypoxic conditions, miRNAs regulate multiple aspects of angiogenesis in PC, providing potential targets and new ideas for further exploration of anti‐angiogenic therapies targeting the hypoxic microenvironment.

In summary, PC angiogenesis is regulated by multiple factors, with miRNAs playing a significant role in both promoting and inhibiting angiogenesis. miRNAs regulate tumour angiogenesis by directly targeting proangiogenic factors such as VEGF or indirectly modulating relevant signalling pathways. A hypoxic microenvironment further complicates this regulatory network by influencing miRNA levels and functions, thereby affecting tumour angiogenesis and malignant progression. While current research has revealed the regulatory potential of miRNAs, it has largely been confined to in vitro settings or mouse models, with insufficient evidence of their clinical translation. Future studies should employ organoid or humanised models to simulate the TME and dissect the dynamic regulatory network of miRNAs, providing new directions for precise anti‐angiogenic therapy.

## 4 INTERPLAY BETWEEN miRNAs AND CAFs IN THE PROGRESSION OF PC

CAFs are highly heterogeneous and stromal cells with dynamic plasticity that are essential for the TME. Through the release of several substances and the alteration within the extracellular matrix, CAFs encourage proliferation and metastasis, and they are closely linked to tumour angiogenesis. Moreover, CAFs can secrete immunosuppressive factors, thereby facilitating tumour immune evasion.^102^ Although CAFs are typically regarded as pro‐tumourigenic in the TME, recent studies have revealed their potential tumour‐suppressive functions. Tumour‐suppressing CAFs (rCAFs) can promote anti‐tumour immunity, form barriers to prevent tumour metastasis and release tumour‐suppressive signals.^103^

Research indicates that exosomes secreted by CAFs carry specific miRNAs and deliver them to PC cells, thereby promoting cancer progression. Research has shown that miR‐21 from CAFs is strongly associated with a bad result in PC patients. It is transported into tumour cells through exosomes, boosting their growth and therapeutic resistance.^104^ Likewise, exosomal miR‐421 from CAFs is transported to cancer cells and accelerates the PC progression by altering the SIRT3/H3K9Ac/HIF‐1α, which is linked to a bad prognosis for patients.^105^ Additionally, under hypoxic conditions, CAFs up‐regulate miR‐21 expression through HIF‐1α/miR‐21. Exosomal miR‐21 from CAFs improves the drug resistance of PC cells by triggering RAS/AKT/ERK.^106^ Upon GEM treatment, CAFs release exosomes loaded miR‐21, miR‐181a, miR‐221, miR‐222 and miR‐92a, which, by inhibiting PTEN expression, further encourage tumour cell proliferation and medication resistance.^107^ Exosomal miR‐125b‐5p from CAFs significantly enhances the PC development via decreasing APC.^108^ CAFs release exosomes including miR‐3173‐5p and miR‐106b, which impart GEM resistance through inhibiting ferroptosis‐related genes ACSL4 and TP53INP1, respectively.^109^, ^110^ Exosomes secreted by CAFs also carry miR‐3173‐5p and miR‐106b, which suppress the ferroptosis‐related genes TP53INP1 and ACSL4 to give PC cells GEM resistance, respectively.^111^ Moreover, CAF‐derived exosomes deliver leptin, which increases miR‐224‐3p levels, thereby suppressing ABL2 expression and enhancing PC aggressiveness.^112^ Conversely, PC cells can also modulate CAF function through exosomal miRNA secretion. For example, PC cells secrete miR‐155‐rich microvesicles that convert normal fibroblasts into CAFs, a process dependent on miR‐155‐mediated targeting of TP53INP1.^113^

**FIGURE 4:**
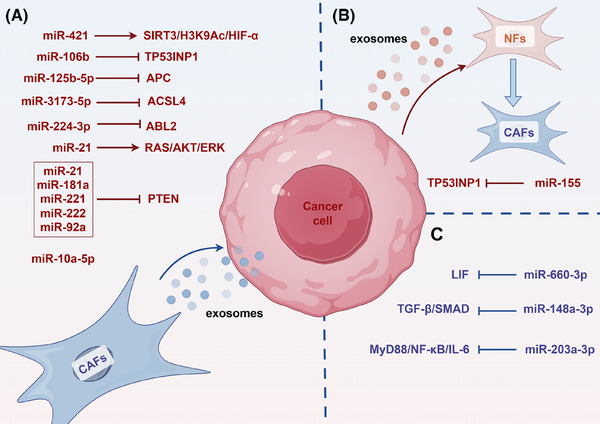
Interplay between miRNAs in PC cells and CAFs. (A) Exosomal miRNAs from CAFs modulate oncogenic pathways in PC cells. (B) Tumour‐secreted exosomal miRNAs reprogrammed NFs into CAFs. (C) Regulatory feedback loops involving cytokine and signalling pathways among CAFs and PC cells. Red text indicates that miRNAs promoting the advancement of PC, whereas blue text denotes miRNAs that suppress tumour development.

In addition to exosome‐mediated mechanisms, through additional pathways, miRNAs affect the part CAFs play in the PC development. miR‐148a‐3p in CAFs suppresses their pro‐tumourigenic activity by targeting ITGA5 and modulating TGF‐β/SMAD.^114^ miR‐203a‐3p in CAFs inhibits their phenotypic transition into inflammatory CAFs via the MyD88/NF‐κB/IL‐6 axis, subsequently reducing IL‐6 secretion and suppressing tumour progression. Furthermore, miR‐660‐3p in CAFs down‐regulates LIF expression, causing the STAT3 signalling pathway to become inactive, which reduces the susceptibility to drugs^115^, ^116^ (Figure 4).

The above studies show that CAFs use exosomes carrying specific miRNAs to interact with tumour cells, which are crucial for the PC development and drug resistance. miRNAs can directly impact the tumour‐promoting ability of CAFs. miRNAs not only facilitate critical signalling between CAFs and tumour cells but also regulate gene expression to affect the TME and tumour cell behaviour. However, present study has mostly focused on the association among miRNAs and tumour‐promoting CAFs, while the study of rCAFs remains underexplored.

## 5 DUAL FUNCTIONS OF miRNAs IN THE MANAGEMENT OF IMMUNE CELLS IN PC

As important elements of the TME, immune cells perform two distinct functions: they can inhibit tumour growth through immune responses, but they may be reprogrammed by the cancer to promote its development. miRNAs are essential for controlling immune cell activity in studies about PC. Many investigations have demonstrated miRNAs affect the polarisation of tumour‐associated macrophages (TAMs), the T cell activity and the functions of DCs, NK cells and myeloid‐derived suppressor cells (MDSCs, thereby contributing to PC's development and immune evasion.

### 5.1 Interactions between miRNAs and TAMs

TAMs are important immune regulatory cells in the TME and have strong plasticity. TAMs are usually divided into two types: the M1‐type with proinflammatory and anti‐tumour functions, and the M2‐type with anti‐inflammatory and pro‐tumour functions.^117^ M2‐type TAMs support tumour progression by secreting immunosuppressive factors and promoting angiogenesis, whereas M1‐type TAMs have the potential to kill tumour cells. The dynamic balance among the two types TAMs plays an essential role to the incidence and progression of malignancies.^118^ Research has shown that the M2‐type macrophages can be reprogrammed into the M1‐type via miRNAs, thereby enhancing anti‐tumour immunity. miR‐155 and miR‐125b can effectively achieve M1‐type polarisation of macrophages through nanoparticle delivery systems, offering fresh perspectives on PC treatment.^119^, ^120^ Similarly, miR‐340 not only enhances the phagocytic capacity of macrophages but also promotes M1‐type polarisation and enhances the amount of CD8^+^ T cells, significantly improving anti‐tumour effects.^121^ miR‐506 can also reprogram TAMs, promoting M1‐type polarisation and enhancing anti‐tumour immunity.^122^ In addition, treatment with apigenin reduces miR‐155 and increases SHIP‐1, raising the percentage of M1‐type macrophages and thereby enhancing the anti‐tumour immune response.^123^

Conversely, miRNAs can also significantly exacerbate the PC malignant progression through encouraging the M2‐type macrophages’ polarisation. M2‐type polarisation is promoted by miR‐124 by regulating the Notch signalling pathway, secreting cytokines such as IL‐6 to activate STAT3, promoting the EMT.^124^ Controlling the secretion of IL‐33, miR‐548t‐5p improves PC cells’ migratory and invasive potential and encourages the activation of macrophages towards the M2 phenotype.^125^ In PC cells, miR‐301 can encourage the polarisation of macrophages towards the M2‐type, thereby enhancing and immunosuppression.^126^ Similarly, miR‐301a‐3p induced under hypoxic conditions is taken up by macrophages through exosomes, activating PTEN/PI3Kγ, driving M2‐type polarisation and enhancing tumour cell migration, invasion and EMT.^127^ Macrophages receive miR‐155‐5p from extracellular vesicles produced from PC cells, which encourages polarisation towards the M2 phenotype.^128^ M2‐type macrophage polarisation can also be aided by extracellular vesicles generated from PC cells that carry miR‐510 and miR‐155‐5p, accelerating the PC development.^128^, ^129^ Furthermore, exosomes produced from PC stem cells (CSCs) that contain miR‐210 can increase resistance to GEM and encourage macrophage polarisation towards the M2 phenotype.^130^ Furthermore, the lncRNA LINC00511 regulates PLAU through a miR‐193a‐3p‐dependent mechanism, driving M2‐type macrophage polarisation and reshaping cancer cell metabolism, thereby increasing their migration and invasion capabilities.^131^ In PC, the lncRNA SNHG17 releases PGK1 mRNA by adsorbing miR‐628‐5p and activating its pro‐tumour function, further promoting M2‐type macrophage polarisation and tumour progression.^132^

Immune cells, such as M2‐type macrophages, can also influence tumour progression through exosomes. For example, miR‐122‐5p in PC can be inhibited by the lncRNA SBF2‐AS1 in exosomes of M2‐type macrophages, thereby inhibiting ability to promote PC development.^133^ miR‐21‐5p in exosomes of M2‐type macrophages targets KLF3 to promote the activity of CSCs, offering a fresh possible target for PC therapy.^134^ In addition, exosomes from M2‐type macrophages carry miR‐222‐3p, which targets the TSC1 gene and increases PC's resistance to GEM.^135^ Exosomes produced from M2‐type macrophages contain miR‐193b‐3p, which can increase the malignant activity of PC cells.^136^ These investigations demonstrate the dual function of miRNAs in controlling the polarisation of macrophages within the immunological milieu of PC.

### 5.2 Role of miRNAs in regulating T‐cell function

In the TME, CD8^+^ T cells and CD4^+^ T cells are two important subgroups of T cells that are essential for anti‐tumour immunity. CD8^+^ T cells directly kill tumour cells, thereby exerting anti‐tumour effects.^137^ CD4^+^ T cells play dual roles: they help CD8^+^ T cells become more cytotoxic, and their regulatory T cells (Tregs) release immunosuppressive substances to prevent CD8^+^ T cells from being active, which accelerates the growth of tumours. The dual function of miRNAs in either boosting or decreasing CD8^+^ T cell anti‐tumour activity is the main focus of current study.^138^ Overexpression of miR‐194‐5p can decrease the growth of PC by encouraging CD8^+^ T cell infiltration and enhancing their capacity to produce IFN‐γ.^139^ Additionally, miR‐429 reduces the immunosuppressive effects of Tregs, enhancing the function of CD8^+^ T cells.^140^ miR‐503‐5p can also strengthen CD8^+^ T cells, but LINC00460 can suppress it through a ‘sponge effect’.^141^ However, certain miRNAs weaken T cell functions by different mechanisms. Exosomal miR‐155‐5p from PC cells can lead to the suppression of CD8+ T‐cell function.^128^ These discoveries demonstrate the two distinct functions of miRNAs in positively and negatively regulating T‐cell function.

### 5.3 Interactions between miRNAs and DCs

PC cells influence the activation and antigen‐presenting capacity of DCs through miRNAs. miR‐203 and miR‐212‐3p can be delivered from PC cells to DCs via exosomes, which down‐regulate TLR4 and MHC II, respectively, leading to impaired DC function and further promoting tumour immune evasion.^142^, ^143^ In contrast, miR‐128 and miR‐194‐5p can enhance the DC function through regulating the ZEB1/CD47 axis or B7‐H1 expression, enhancing immune responses.^144^, ^145^

### 5.4 Regulation of NK cell and MDSC functions by miRNAs

Studies have demonstrated that miRNAs influence PC immune evasion by controlling NK cells’ cytotoxicity and immune surveillance capacities. miR‐4299 affects the immunological surveillance of NK cells towards PC by controlling ADAM17/MICA/B,^146^ while the down‐regulation of miR‐1275 under hypoxic environments weakens the cytotoxicity of NK cells.^147^ Furthermore, NK cells can deliver let‐7b‐5p to PC cells via exosomes, and by specifically targeting CDK6, it prevents the growth of cancer cells.^148^ MDSCs are also important components of PC immune evasion. miR‐494‐3p and miR‐1260a influence the calcium ion influx of MDSCs through PC‐derived exosomes, affecting their immunosuppressive activity.^149^

### 5.5 Regulation of immune cell proportions by miRNAs

**FIGURE 5:**
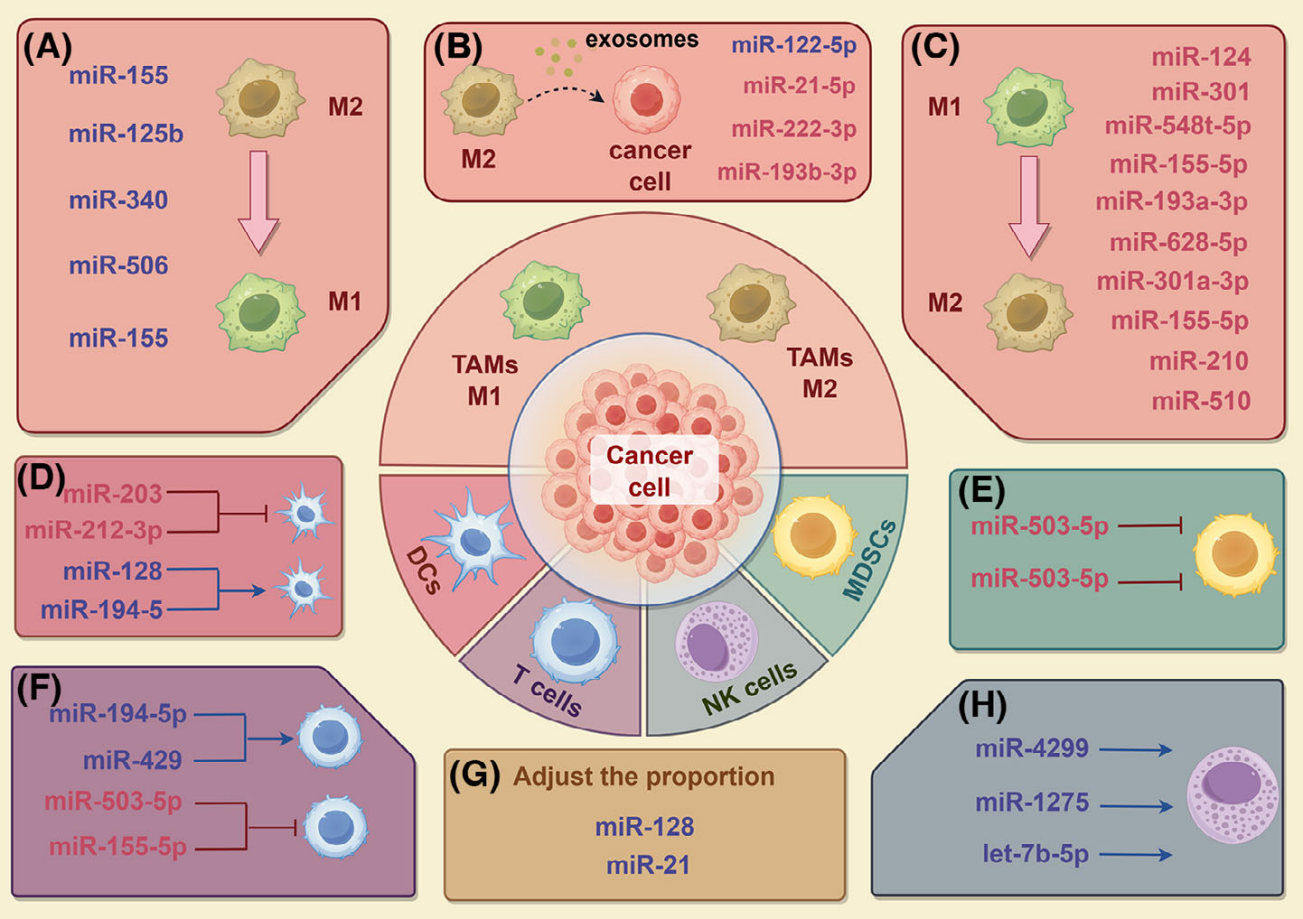
Dual functions of miRNAs in the management of immune cells in PC. (A) Key miRNAs involved in inducing TAMs to repolarise from the pro‐tumour M2‐type to the anti‐tumour M1‐type, thereby enhancing anti‐tumour immunity. (B) Exosomal miRNAs derived from M2 macrophages influence PC progression. (C) Key miRNAs involved in inducing TAMs to repolarise from the anti‐tumour M1‐type to the pro‐tumour M2‐type, thereby promoting PC development. (D) miRNA‐mediated modulation of DC activation and antigen presentation. (E) miRNAs governing MDSC activity. (F) miRNAs that potentiate or attenuate T‐cell anti‐tumour activity. (G) miRNAs that influence immune cell proportions. (H) miRNAs governing NK cell function. Red text indicates that miRNAs weaken anti‐tumour immunity, whereas blue text denotes that miRNAs enhance anti‐tumour immunity.

An analysis of the immune microenvironment revealed that the overexpression of miR‐128 in tumour cells increases the ratios of NK cells, CD8^+^ T cells and DCs in the spleen and tumour, thereby enhancing the anti‐tumour immune response.^144^ miR‐21 contributes significantly to the immunosuppressive microenvironment of PC, which increases the proportion of MDSCs, Tregs and M2‐type macrophages, providing a new target for future immunotherapy strategies.^150^ In addition, four miRNAs (hsa‐miR‐3178, hsa‐miR‐485‐3p, hsa‐miR‐574‐5p and hsa‐miR‐584‐5p) are connected to drug‐resistant PC cells and affect the localisation of CD4^+^ memory T‐cell subsets, significantly impacting patient prognosis (Figure 5).^151^

These studies indicate that although different miRNAs have various mechanisms of action in regulating immune cells, overall, miRNAs can influence the dynamic balance of the immune microenvironment through multiple mechanisms, including direct targeting of signalling molecules or through intercellular communication mediated by exosomes. These studies demonstrate the miRNAs’ variety and intricacy in the immune regulation of PC and suggest that controlling immune responses via miRNAs has great promise for treatment. However, the present study still has a few constraints. The immune cell subpopulations’ high heterogeneity makes it difficult to precisely dissect the targeting effects of miRNAs. In the future, single‐cell transcriptomics, spatial metabolomics and epigenetics should be combined to map the interactions between immune cells and miRNAs.

## 6 FUNCTIONS OF miRNAs IN THE NEURAL MICROENVIRONMENT

Nerves are also important components of the TME and have multifaceted effects on the incidence and progression of cancers.^152^ Neurons and their projections (axons) can serve as inducible characteristic components in the TME, significantly influencing tumour growth and spread.^153^ Axons not only promote tumour proliferation but are also closely related to tumour‐derived nerve growth factor (NGF). NGF initiates the expansion of sensory afferent and autonomic efferent nerve fibres around the tumour by stimulating axonal growth, thereby further enhancing the interaction between nerves and tumours. These nerves that innervate the tumour release specific molecular signals, directly regulating the development of cancers and forming a complex tumour‐nerve interaction network.^154^ In recent years, as research has increasingly shown, miRNAs play a crucial function in the neuronal microenvironment of PC, revealing specific mechanisms and potential clinical significance. NGF enhances the perineural invasion (PNI) capability of tumour cells by promoting the expression of exosomal miRNA‐21‐5p secreted by PC cells.^155^ In PC, the Hedgehog‐Gli1 mediates neural restructuring via controlling the miR‐451a/VGF through exosomal circ0011536.^156^ In addition, the exosomal lncRNA XIST significantly promotes PNI in PC cells through the miR‐211–5p/GDNF axis,^157^ further emphasising the importance of miRNAs in regulating the interaction between tumours and the neural microenvironment.

## 7 FUNCTIONS OF miRNAs IN THE METABOLIC REPROGRAMMING

Additionally, the development of malignancies are significantly influenced by metabolic reprogramming. The metabolism of tumour cells are not only driven by intrinsic factors but also regulated by metabolites in the TME.^158^ Metabolic reprogramming allows tumour cells to adapt, thrive and multiply in the nutrient deficient TME. Oncogenes and tumour suppressor genes are typically activated or mutated to drive this process, which alters the expression and activity of important metabolic enzymes in cellular metabolic signalling networks.^159^ miRNAs, which regulate key metabolic enzymes, signalling pathways and interactions with ceRNAs, constitute a core element of the metabolic regulation network.

Many studies have centred on the direct targeting key glycolytic enzymes via miRNAs. miR‐135 reduces the glycolytic capacity of PC cells by inhibiting phosphofructokinase‐1.^160^ miR‐489‐3p^161^, ^162^, ^163^ targets pyruvate kinase M2 (PKM2), significantly reducing glycolytic activity while also reducing the tumour cells’ capacity for invasion and multiplication. Additionally, miR‐489‐3p^162^ inhibits glycolysis by targeting lactate dehydrogenase A (LDHA). Notably, many miRNAs focus on regulating hexokinase 2 (HK2), a key upstream enzyme in glycolysis. For example, miR‐202,^164^ miR‐323a,^165^ miR‐125b‐5p,^166^ miR‐185‐5p,^167^ miR‐5586‐5p^168^ and miR‐330‐5p target HK2, reducing glycolytic levels and preventing PC cells from proliferating and spreading. Moreover, miR‐200c‐3p,^169^ miR‐769‐3p^170^ and miR‐326‐3p^171^ inhibit key glycolytic enzymes by inactivating PI3K/AKT/mTOR, ultimately suppressing glycolysis. Moreover, miR‐590‐5p regulates glucose metabolism reprogramming by targeting Tiam1, thereby modulating the development of cancer cells.^88^

miRNAs influence cellular interactions by regulating metabolism. In PC, miR‐21 prevents the malignant progression through suppressing the glycolytic activity of PSCs, thereby reducing their migration and invasion capabilities.^172^ LINC00511, after being transferred via exosomes, influences macrophage polarisation to the M2 type via miR‐193a‐3p‐dependent modulation of PLAU while also enhancing glycolysis, further encouraging PC cells to proliferate and migrate.^131^

**FIGURE 6:**
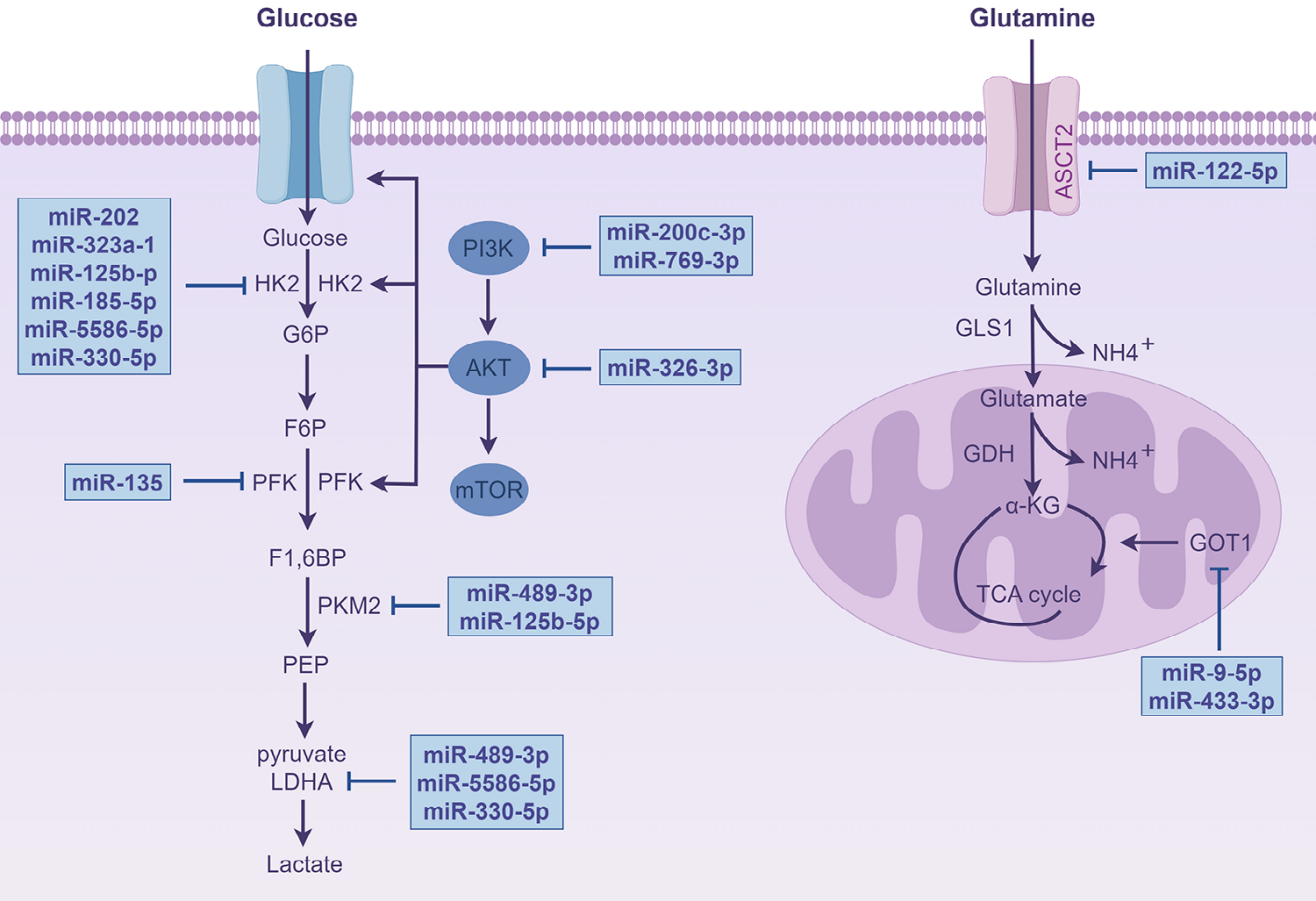
Regulation of glycolysis and glutamine metabolism by miRNAs. miRNAs exert coordinated regulation over critical metabolic enzymes, including HK2, PFK, PKM2 and LDHA, in glycolysis, as well as ASCT2 and GOT1 in glutamine metabolism, while interfacing with central signalling pathways. The text in the blue box indicates miRNAs that suppress tumour development.

In addition to glycolysis, miRNAs are involved in other metabolic pathways. In PC, palbociclib activates AMPKα by increasing the level of miR‐33a, inhibiting fatty acid generation and enhancing apoptosis.^173^ circ_03955 regulates the expression of HIF‐1α by adsorbing miR‐3662, promoting pancreatic tumour formation and the Warburg effect.^174^ Furthermore, miR‐9‐5p and miR‐122‐5p target GOT1 and ASCT2, respectively, to inhibit glutamine metabolism.^175^, ^176^ Moreover, circ‐MBOAT2 controls glutamine metabolism via the miR‐433‐3p/GOT1, offering additional possible biomarkers for PC detection and treatment (Figure 6).^177^

According to the studies described above, miRNAs are important regulators of PC's metabolic reprogramming, not only by directly targeting metabolic enzymes but also by interacting with ceRNAs and modulating signalling pathways, affecting key processes such as glycolysis, fatty acid production and glutamine metabolism. These processes stimulate the growth and invasion of PC and make them to adapt to the nutrient‐deprived TME through metabolic reprogramming, thereby promoting tumour progression.

## 8 DISCUSSION

The critical functions of miRNAs in the PC TME are well outlined in this review. miRNAs modulate angiogenesis, interact with CAFs to remodel the matrix, regulate immune cell functions and proportions, mediate exosome‐based signalling, serve as biomarkers, shape the neuro‐microenvironment and drive metabolic reprogramming, all of which significantly impact PC progression. These results highlight the potential uses of miRNAs in detection and treatment in PC.

However, current research on miRNAs in the PC TME has focused mainly on individual miRNAs. The complex regulatory network and synergistic effects of miRNAs in the PC TME are not yet fully understood. Given the dynamic and complex nature of the TME, the roles and mechanisms of miRNAs in different cell types remain to be elucidated. Future studies should integrate single‐cell sequencing and high‐throughput omics technologies to investigate the dynamic changes and mechanisms of miRNAs across different regions and cell types precisely.

Interestingly, in the PC TME, the functional plasticity of miRNAs manifests not only as the continuation of the same function but also as the coexistence of opposite functions. Some miRNAs show functional consistency in different aspects of the TME. For example, miR‐21 forms a multidimensional procancer network by promoting angiogenesis, enhancing CAF‐mediated drug resistance, regulating the proportion of immune cells and driving nerve infiltration. On the other hand, the same miRNA may lead to functional contradictions due to differences in target genes or conflicts in regulatory pathways. This functional diversity may stem from conditioned functional changes and the cross‐regulation of signalling pathways. miR‐301a promotes angiogenesis under normoxic conditions, whereas in the hypoxic microenvironment, it drives the M2 polarisation of macrophages through exosomes, and its functional conversion depends on environmental pressure. On the one hand, miR‐200c promotes angiogenesis by up‐regulating VEGF. On the other hand, it restricts glycolysis by inhibiting PI3K/AKT/mTOR, implying a dual function of preventing and causing cancer. This dynamic plasticity indicates that miRNAs are not merely simple cancer‐promoting or cancer‐suppressing molecules. Instead, it integrates multiple signals in the TME and interacts specifically with specific targets to form a complex regulatory network, therefore playing a diverse regulatory function in the development, occurrence and reconfiguration of the PC TME.

Nevertheless, there are a number of obstacles to the clinical use of miRNAs about PC treatment. miRNAs have poor stability and are prone to rapid degradation in the blood circulation, resulting in a short half‐life and compromised therapeutic efficacy.^178^, ^179^ Moreover, miRNA transport is hampered by the high interstitial fluid pressure and thick extracellular matrix found in tumour tissues, especially the fibrotic microenvironment of PC.^180^ In addition, systemic miRNA delivery may trigger innate immune responses, causing adverse side effects.^178^ The off‐target effects of miRNAs can also lead to non‐specific gene silencing, potentially causing toxicity or reducing therapeutic effects.^180^ To address these issues, researchers have developed various delivery strategies. Oncolytic viruses can target miRNA delivery, increase tumour specificity or use miRNA to increase adenoviral oncolytic activity.^181^, ^182^ Through functionalisation, nanoparticles can improve delivery accuracy, efficiency and penetration.^183^, ^184^ Liposomes protect miRNAs, increasing their stability and promoting their cellular uptake.^185^ Exosomes utilise natural vesicles in the TME to mediate intercellular communication and regulate key signalling pathways.^94^, ^109^ However, current delivery systems still have limitations and cannot resolve all the problems associated with miRNA application. Future research should focus on developing smart responsive delivery systems, such as carriers sensitive to pH and enzyme activity, to achieve spatiotemporally controlled miRNA release and reduce off‐target effects and toxicity. Multiple collaborative delivery methods can be integrated to develop carriers for loading miRNAs with chemotherapy drugs or immunomodulators. Furthermore, combining single‐cell sequencing and spatial transcriptomics to analyse PC heterogeneity can guide precise delivery system interventions for specific cell subpopulations. These approaches can advance miRNA therapy towards greater precision and efficiency.

## AUTHOR CONTRIBUTIONS

Jie Ji contributed to the research conception and design. Jie Ji, Dandan Jin and Junpeng Zhao drafted the manuscript and counted and plotted the diagrams and tables. Xudong Xie, Yujie Jiao, Xuyang He, Yuxuan Huang and Lirong Zhou embellished and modified the contents and diagrams of the manuscript. Mingbing Xiao and Xiaolei Cao critically revised the article for important intellectual content. All the authors have read and approved the submitted manuscript.

## CONFLICT OF INTEREST STATEMENT

All the authors declared that they have no potential conflicts of interest with respect to the research, authorship or publication of this article.

## ETHICS STATEMENT

The authors have nothing to report.

## Data Availability

No datasets were generated or analysed during the current study.
